# Diuretic Activity of the Aqueous Extract Leaves of *Ficus glumosa* Del. (Moraceae) in Rats

**DOI:** 10.1155/2014/693803

**Published:** 2014-10-14

**Authors:** Fidèle Ntchapda, Djedouboum Abakar, Blaise Kom, Paulin Nana, Christian Bonabe, Maguirgue Kakesse, Emmanuel Talla, Théophile Dimo

**Affiliations:** ^1^Department of Biological Sciences, Faculty of Science, University of Ngaoundéré, P.O. Box 454, Ngaoundéré, Cameroon; ^2^Department of Chemistry, Faculty of Science, University of Ngaoundéré, P.O. Box 454, Ngaoundéré, Cameroon; ^3^School of Wood, Water and Natural Resources, Faculty of Agriculture and Agricultural Sciences, University of Dschang, Ebolowa Campus, P.O. Box 786, Ebolowa, Cameroon; ^4^Department of Animal Biology and Physiology, Faculty of Science, University of Yaoundé I, P.O. Box 812, Yaoundé, Cameroon

## Abstract

Experiments were carried out to validate the use of *F. glumosa* extract as a diuretic in the treatment of hypertension as claimed by traditional healers. The experiments were performed under the same conditions with two synthetic pharmacological diuretics considered as check (Furosemide and Amiloride hydrochlorothiazide). The aqueous extract leaves of *F. glumosa* accelerated the elimination of overloaded fluid. At the maximum of diuretic response, urinary osmolarity decreased significantly when compared with controls. The single dose treatment of the aqueous extract leaves of *F. glumosa* has significantly increased urine volume 24 h after administration of the extract. The stability of aldosterone level, the absence of correlation with the plasma levels of sodium, and the increased clearance of free water in the animals receiving the extract show that increased diuresis and natriuresis moderate elevation are tubular in origin. The increase in Na^+^, K^+^, and Cl^−^ induced by the extract caused alkalinization of the urine and showed a strong inhibitory effect of carbonic anhydrase and saluretic. These effects were mainly observed at the dose of 375 mg/kg. These observations confirm the traditional use in the treatment of hypertension and support the importance of the conservation of local knowledge as well as the conservation of Cameroonian biodiversity.

## 1. Introduction

The overuse in automedication of phytotherapeutic preparations is the main means to cure about 80% population who is unable to get access to manufactured drugs [1]. High blood pressure represents an important risk factor to development of other cardiovascular diseases and constitutes one of the main causes of mortality in the world [1]. Among the important diseases, hypertension plays a key role due to its impact on survival. Many control systems can be modulated by plant metabolites [2]. Regardless of their specific pharmacological effects, most medicinal plants have been described as having a more or less pronounced diuretic effect. However, very few studies have examined the mechanisms of action of inducing renal excretion.* Ficus glumosa* (Moraceae) is a small- to medium-sized tree, 5–10 m tall. Branches are widely spread, more or less horizontal, often supported by stilt roots. This plant is used in traditional medicine in Cameroon, Senegal, and East Africa for the treatment of edema, hypertension, diabetes, hemorrhoids, rheumatism, and stomatitis [3, 4]. The leaves are also used in these regions for the treatment of skin diseases and diabetes [5]. Ficus species are known for their content of furanocoumarins. The biological properties of these molecules require in the majority of the cases an exposure to UV. [6, 7]. One of the fundamental properties of the furocoumarins is the photocycloaddition with the nucleic acids. In molecular biology, from their capacity to fix and induce breaks in DNA, the furocoumarins constitute a practical tool for the analysis of the mechanisms of repair of ADN, like for the study of chromatin [8]. Pharmacological properties of* F. glumosa* extracts on gastrointestinal motility were demonstrated in 2012 by Tanko et al. [9]. Umar et al. [10] also showed the effects of ethanol leaf extract of* Ficus glumosa* on fasting blood glucose and serum lipid profile in diabetic rats. Leaves of this tree are also used to cook porridge in Far North of Cameroon and southern Chad. Decoction of the leaves is used as a galactogenic for both women and female animals [11]. Phytochemical screening of this plant revealed the presence of flavonoids, saponins, carbohydrates, tannins, and triterpenes [9]. The present work aims at measuring the diuretic power of* F. glumosa* in acute treatment.

## 2. Materials and Methods

### 2.1. Plant Materials

The leaves of* F. glumosa* were harvested in Ngaoundéré Adamawa region of Cameroon. Then we proceeded to the identification by comparing the harvested plant to specimen no. 60695/HNC deposited at the National Herbarium of Cameroon.

### 2.2. Animals

Wistar rats (250–350 g) of both sexes were used in all experiments. Strains of animals were from Centre Pasteur in Yaoundé. They were reared in the Department of Biological Sciences, Faculty of Sciences (University of Ngaoundéré). The animals were housed under controlled temperature (24 ± 2°C) and relative humidity (45% ± 10%). Moreover, they had free access to food (pellets from LANAVET (Laboratory NVS)) and tap water. The animal handling was under the control of the veterinary surgeon of the Science Veterinary Surgeon and Medical School of the University of Ngaoundéré. Experimental protocols and procedures were approved by the Institutional Animals Care and Use Committee and the research was approved by the Animal Ethics Committee of the University of Ngaoundéré.

### 2.3. Preparation of the Aqueous Extract of* Ficus glumosa* Del

One thousand grams of fresh leaves of* F. glumosa* was steeped in 1 L of distilled water for 12 hours at room temperature. The macerate was filtered through Whatman filter paper No. 3, and the filtrate was concentrated in a rotary evaporator at 40°C for 24 hours. This process repeated several times yielded 11,2 g of concentrated crude extract in the form of an oily paste. The extract was stored at −20°C.

### 2.4. Antidiuretic Activity

In preliminary tests, 1 mL/100 g body weight of distilled water was administered to rats. They were then individually placed in metabolic cages where urine was collected and the volume was measured after 6 hours. The animals excreting at least 40% of the volume of dosing solution were selected for tests. Those having less than 40% excretion were excluded [1]. The selected rats were divided into 5 groups of 5 rats each. The animals were then placed individually in metabolic cages for acclimatization for 7 days. The aqueous extract of* F. glumosa* at increasing doses of 75, 150, 225, 300, and 375 mg/kg was administered to animals at 0.5 mL/100 g body weight. Urine volume was determined 1, 2, 3, 4, 5, 6, and 24 hours after administration of the extract. The animals of control groups were treated with distilled water, Furosemide (2 mg/kg), and Amiloride hydrochlorothiazide (14 mg/kg).

### 2.5. Preparation of Kidney Homogenates

The animals were sacrificed by decapitation at the end of the experiment. The arteriovenous blood was collected in heparinized tubes and centrifuged. The plasma collected was stored at −20°C for biochemical analysis. The kidney was removed, cleaned of fat material, weighed, and stored at 20°C for biochemical analyzes.

### 2.6. Parameters

Urinary and plasma concentrations of sodium and potassium ions were determined using flame photometry (Jenway, PFP 7) according to standard methods [12]. Concentrations of creatinine, urea, aldosterone, glucose, albumin, and electrolytes in the plasma and urine samples were evaluated using a two-way digital spectrophotometer (Secomam). In animals treated with the extract and reference substances, urinary osmolarities and natriuresis were measured during the diuretic response, particularly when at the maximum excretion rate. The osmolarity of plasma and urine samples was measured by cytometry using an osmometer (Knauer). The aldosterone was measured by radioimmunoassay (assay kit Aldo RIACT). Osmolar clearance (Cosm) was determined from plasma osmolality (POSM), urinary osmolarity (Uosm), and urine flow (V) according to the following formula: Cosm = Uosm. V/POSM. When the solutes are removed in a larger quantity of water than filtered plasma volume water, free water clearance (C_H_2_O_ = V − Cosm) is positive. Glomerular filtration is determined from the clearance of creatinine. The GFR (glomerular filtration rate) was assessed by creatinine clearance (CreatC). The amount of Na^+^ and K^+^ was calculated as a parameter for the saluretic activity. The ratio of Na^+^/K^+^ was calculated for the natriuretic activity. To estimate the carbonic anhydrase inhibition activity, the ratio of Cl^−^/(Na^+^ + K^+^) was calculated [13].

### 2.7. Phytochemical Study

Analytical tests for the identification of different families of metabolites in crude extracts of the leaves were made at IMPM (Institute of Medicinal Plants for Medicinal research), Cameroon. The procedures described in 1983 by Trease and Evans [14] were used for the detection of various chemical groups. In view of the identification of the chemical structure of the compounds responsible for the diuretic activity, preliminary tests of the phytochemical study were conducted.

### 2.8. Statistical Analyses

The results expressed are the mean ± SEM (*n* = 5). Comparison of means was made using the Student's *t*-test and one-way ANOVA of Origin Graph software (Microcal Origin 6.0), software version 6.0. *P* < 0.05, *P* < 0.01, and *P* < 0.001 difference was considered significant.

## 3. Results

### 3.1. Phytochemical Study

Phytochemical screening performed on crude extracts revealed the presence of several primary and secondary metabolites such as fatty acids, athraquinones, glycosides, saponins, tannins, coumarins, and triterpenes. Phenolic compounds and sterols are also present in the extract. The presence of flavonoids and alkaloids is remarkable. These initial results suggest that the aqueous extract of leaves of* Ficus glumosa* contains several chemical compounds whose potential biological activity remains to be demonstrated.

### 3.2. Diuretic Activity

#### 3.2.1. Kinetic of Hydroelectrolytic Eliminations

**Figure 1:**
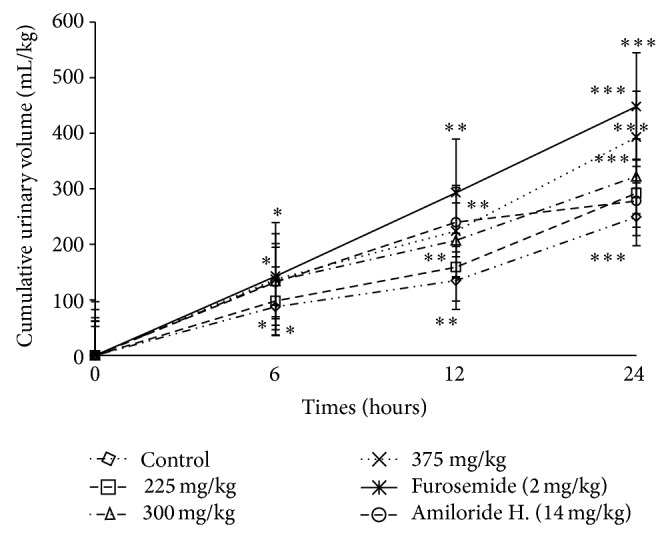
Effects of the aqueous extract of* F. glumosa* on the cumulative urinary volume excretion. Values are means ± SEM, *n* = 5, ∗ < 0.05, ∗∗ < 0.01, and ∗∗∗ < 0.001, significant difference compared to the control.

**Figure 2:**
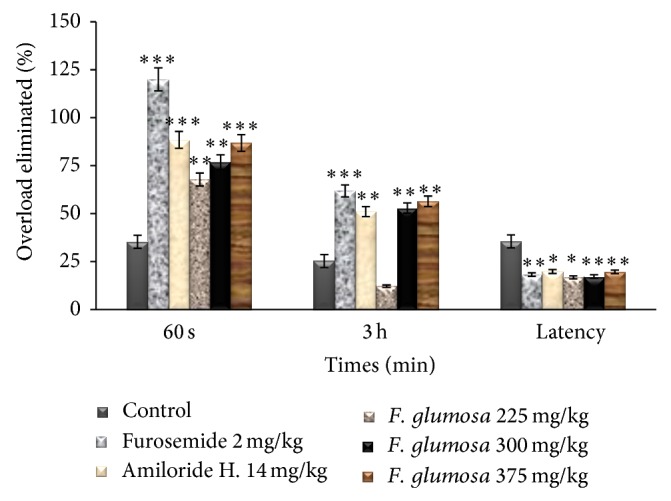
Effects of the aqueous extract of* F. glumosa* on the overload eliminated. Values are means ± SEM, *n* = 5, ∗ < 0.05, ∗∗ < 0.01, and ∗∗∗ < 0.001, significant difference compared to the control.

*(1) Effect of Ficus glumosa Extract on the Urinary Volume.* A single dose-response administration of the aqueous extract of* F. glumosa* (75, 150, 225, 300, and 375 mg/kg) significantly increased (*P* < 0.05) the volume of urine 24 hours later. However, the urinary excretion was dose dependant (Table 1). The volume of urine of 215.31 ± 4.93 mL/kg/24 h in controls (distilled H_2_O) significantly increased to 292.15 ± 4.69 mL/kg/24 h (35.68% increment) at the dose of 225 mg/kg. At the dose of 300 mg/kg, the volume of urine increased by 49.70%. The highest dose (375 mg/kg) induced 82.54% increase of the treated group. The volume of urine went from 231.31 ± 2.38 mL/kg/24 h in control group to 393.04 ± 3.62 mL/kg/24 h in the group treated with the highest dose (Figure 1). The results in Figure 2 show clearly the improvement of renal excretion of the overload in the presence of the aqueous extract of* F. glumosa*, Furosemide, and Amiloride hydrochlorothiazide. Aqueous leaf extract of* F. glumosa* accelerates the elimination of fluid overload; the latency of the first urination decreased significantly (*P* < 0.05, *P* < 0.01, and *P* < 0.001) (Figure 2). The latency of the first urination was 32 ± 3 min in controls, 17 ± 2 minutes in animals treated with the aqueous extract of leaves* F. glumosa*, 18 ± 2 min in the group treated with Furosemide, and 18 ± 3 min in the group treated with Amiloride hydrochlorothiazide (Figure 2).

The percentage of overload eliminated in urine was 46.35 ± 3.21% in control and, respectively, 76.87 ± 3.36% and 86.87 ± 5.06% in rats treated with the extract of* F. glumosa* at the dose of 300 and 375 mg/kg. It was 120 ± 4.11% in animals treated with Furosemide and 88.43 ± 4.41% in those treated with Amiloride hydrochlorothiazide (Figure 2). Aqueous extract of the leaves of* F. glumosa* (225, 300, and 375 mg/kg) 3 hours after administration significantly (*P* < 0.05) increased dose-dependently the quantity of urinary excretion. Urine volume increased from 87.44 ± 1.41 mL/kg in controls to 98.14 ± 1.67 mL/kg at the dose of 225 mg/kg, meaning an increase of 12.23% and, respectively, to 133.68 ± 1.22 mL/kg and 136.78 ± 1.08 at doses 300 and 375 mg/kg. The urinary volume of animals treated with Furosemide and Amiloride hydrochlorothiazide meanwhile has increased by 61.87% and 51.04%, respectively (Figure 2).

**Figure 3:**
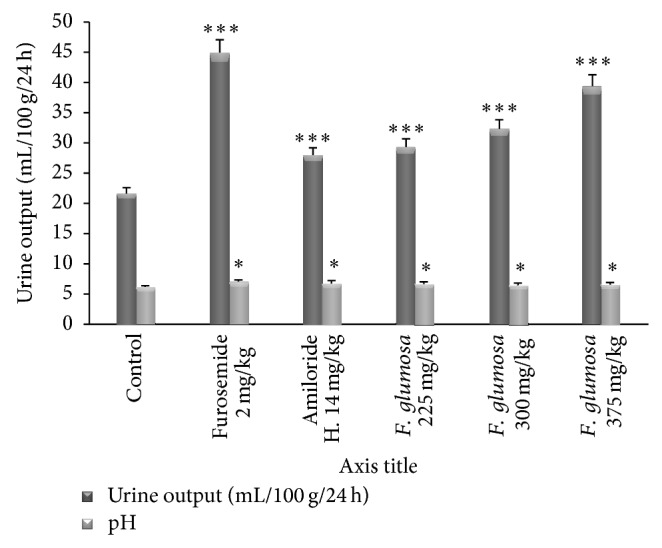
Effects of the aqueous extract of* F. glumosa* on the urine output and pH. Values are means ± SEM, *n* = 5, ∗ < 0.05, ∗∗∗ < 0.001, significant difference compared to the control.

*(2) Effect of Aqueous Extract of F. glumosa on Urine Output Index and pH.* Diuretic index of the plant extract is lower than that of Furosemide and higher than that of Amiloride hydrochlorothiazide at doses 225 mg/kg, 300 mg/kg, and 375 mg/kg (Table 3). At the dose of 225 mg/kg,* F. glumosa* showed a significantly (*P* < 0.05) high urinary pH when compared to the control group (Figure 3). The pH values (6.6 ± 0.1) of urine of those treated with the extract of* F. glumosa* were higher than the control group (6.1 ± 0.2). Doses of 225 and 300 mg/kg showed significantly increased pH values. However, the pH values (6.6 ± 0.1) of urine of animals treated with the extract were lower than that of rats treated with pharmacological substances (7.0 ± 0.1) (Figure 3).

#### 3.2.2. Electrolyte Excretion

**Figure 4:**
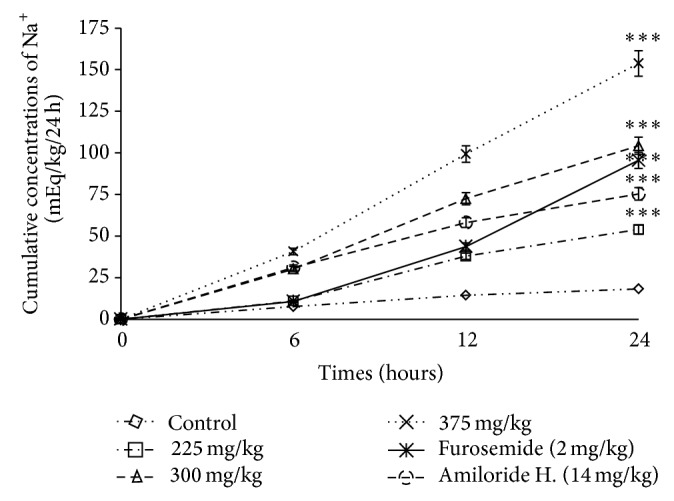
Effects of the aqueous extract of* F. glumosa* on the cumulative Na^+^ excretion. Values are means ± SEM, *n* = 5, ∗∗∗ < 0.001, significant difference compared to the control.

*(1) Effects of F. glumosa on Cumulative Urinary Excretion of Sodium (Na* ^*+*^ *).* The excretion of Na^+^ excretion increase was 53.92 ± 1.78 mEq/kg/24 h for the dose of 225 mg/kg, 104.24 ± 1.76 mEq/kg/24 h for the dose of 300 mg/kg, and 153.78 ± 2.02 mEq/kg/24 h for the highest dose (375 mg/kg) (Table 1). The Amiloride hydrochlorothiazide (14 mg/kg) induced as the extract an increased in sodium excretion (75.30 ± 5.12 mEq/kg/24 h), without significant changes (*P* > 0.05) in urinary K^+^ excretion. Furosemide also induced a significant increase (*P* < 0.05) from 18.27 ± 1.01 mEq/kg/24 h in the control group to 95.45 ± 4.16 mEq/kg/24 h in the treated group (Figure 4).

**Figure 5:**
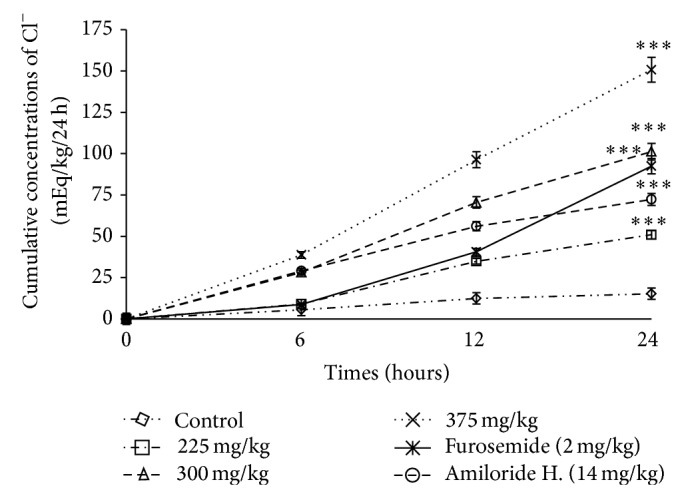
Effects of the aqueous extract of* F. glumosa* on the cumulative Cl^−^ excretion. Values are means ± SEM, *n* = 5, ∗∗∗ < 0.001, significant difference compared to the control.

*(2) Effects of F. glumosa on Cumulative Urinary Excretion of Chlorine (Cl* ^*−*^ *).* Aqueous extract of* F. glumosa* showed a significant increase in the excretion of chloride ions. Excretion increased from 15.27 ± 3.65 mEq/kg/24 h in controls to 50.92 ± 2.35 mEq/kg/24 h at the dose of 225 mg/kg (Table 1). This increase was 562.99% at the dose 300 mg/kg and reached 887.42% at the dose 375 mg/kg. Rats treated with Furosemide and Amiloride hydrochlorothiazide, respectively, excreted 505.43% and 373.47% of chloride ions as compared to controls (Figure 5).

**Figure 6:**
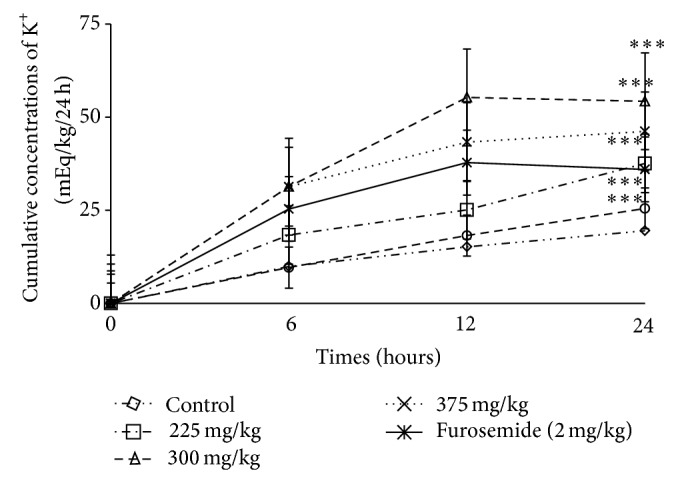
Effects of the aqueous extract of* F. glumosa* on the cumulative K^+^ excretion. Values are means ± SEM, *n* = 5, ∗∗∗ < 0.001, significant difference compared to the control.

*(3) Effect of F. glumosa on Cumulative Urinary Excretion of Potassium (K* ^*+*^ *).* Urinary potassium excretion was decreased to 20.51  ±  1.29 mEq/kg/24 h in animals treated with Amiloride hydrochlorothiazide (14 mg/kg). Furosemide (2 mg/kg) as well as the extract induced an increase of potassium excretion of 65.99  ±  5.62 mEq/kg/24 h (Table 1). At a dose of 225 mg/kg, there was a potassium increase of 37.58 ± 2.52 mEq/kg/24 h, whereas the dose of 300 mg/kg increased potassium to 84.29  ±  5.11 mEq/kg/24 h and the dose of 375 mg/kg increased potassium to 76.21 ± 6.87 mEq/kg/24 h when compared to the control group 19.54 ± 2.88 mEq/kg/24 h (Figure 6).

### 3.3. Saluretic, Carbonic Anhydrase Inhibition, and Natriuretic Activity

Table 2 shows the activity of saluretic, natriuretic, and CAI after administration of the extract and the reference substances. The dose of 375 mg/kg showed a significant increase in saluretic and natriuretic activities when compared to the control group. Increase of 812.45% and 117.20% was, respectively, recorded in saluretic and natriuretic activities. Natriuretic ratio > 2.0 indicates a favorable natriuretic activity [13]. All the three doses of the plant extract showed a dose dependent increase in the value of Na^+^/K^+^, which has proven to be significant at the threshold *P* < 0.05. The extract at a dose 375 mg/kg produced a remarkable value of Na^+^/K^+^. The significant natriuretic ratio (3.33) showed a significant natriuretic activity. A CAI < 0.8 report indicates a strong decrease in diuretic activity [13]. Extract of* F. glumosa* doses mentioned above shows a CAI > 0.8, indicating a significant diuretic activity. The saluretic dose index 375 is considerable. With a value of 9.12, it is 1.62 times larger than that of Furosemide which is 5.63.

### 3.4. Effects of* F. glumosa* on Serum Parameters

Some hematological parameters were evaluated in rats treated with the plant extract and pharmacological substances used as a check. The glycemia of rats ranged between 94.39 ± 10.45 mg/dL and 98.28 ± 7.10 mg/dL in animals in disregard of the treatment. There was a significant increase (*P* < 0.05) of 43.54% and 11.25%, respectively, of creatinine and urea in animals that received the extract at dose 375 mg/kg. The animals receiving Furosemide and Amiloride hydrochlorothiazide also show a significant increase (*P* < 0.05) in the rate of creatinine and urea (25.8% and 6.96% for Furosemide and 22.58% and 15.04% for Amiloride hydrochlorothiazide). Albuminuria increased from 42.8 ± 5.01 g/L in the control group to 44.8  ±  5.06 g/L in animals that received the extract at a dose of 375 mg/kg, meaning an increase of 4.67%. In animals treated with Furosemide and Amiloride hydrochlorothiazide, there was a significant increase (*P* < 0.05) in the rate of albumin, respectively, of 4.67% and 2.10%. The concentrations of Na^+^ and K^+^ ions were significantly increased (*P* < 0.05), respectively, 509.24% and 182.16% in animals that received the extract at the dose of 375 mg/kg (Table 4). The increase in plasma osmolality and aldosterone levels was, respectively, 4.96% and 3.70%, while animals treated with Furosemide and Amiloride hydrochlorothiazide had an increased plasma osmolarity of 5.34% and 4.96%, respectively; the aldosterone levels increased by 3.30% and 0.36%, respectively (Table 4).

### 3.5. Effects of* F. glumosa* on Index Kidney Function

The analysis of the collected urine of rats 24 hours after administration of a single dose of the extract of* F. glumosa* revealed no trace of glucose and albumin. The aqueous extract of* F. glumosa* caused no significant change in rate of urinary creatinine. However, the concentration of urea in the urine was significantly (*P* < 0.05) decreased by 17.91% and 14.48%, at doses of 225 and 300 mg/kg, respectively (Table 5). Osmotic clearance significantly increased by 2.17% at the highest dose. The GFR decreased from 1.60 ± 0.51 mL/min in controls to 1.40 ± 0.14 mL/min (14.28%). The creatinine clearance also decreased by 42.10% (Table 5).

## 4. Discussion

Preliminary phytochemical studies showed that aqueous extract of the leaves of* F. glumosa* contains several chemical compounds that could be partially or fully responsible for the increase of diuresis and moderate natriuretic activity. Results of the study showed an increase and acceleration of the elimination of fluid overload with urinary hypoosmolarity and a moderate increase in natriuretic activity. These results demonstrate that the aqueous extract of the leaves of* F. glumosa* has a moderate diuretic activity. The increase of natriuresis in response to acute treatment by aqueous extract of leaves of* F. glumosa* may partly explain the increase in diuresis [15, 16]. Aldosterone hormone measured by radioimmunoassay was slightly increased in animals treated with aqueous extract (Table 4), and the lack of correlation between plasma aldosterone and sodium concentration in the blood as well as in urine seems to imply that aldosterone is not involved in the natriuresis which observed and suggested that stimulation of diuresis by the aqueous extract of the leaves of* F. glumosa* could be similar to that of Furosemide. The extract as well as Furosemide caused a urinary increase of Na^+^ and Cl^−^ in rats. The increase of the Na^+^ excretion tends to reduce GFR by increasing the Na^+^ load available for Na^+^/K^+^ exchange, stimulating further such exchange by hyperaldosteronism (Table 4), which causes a reduction in blood volume [17, 18].

The increase of Na^+^ in the* macula densa* inhibits renin secretion, which tends to increase the GFR, but the decrease in blood volume increases renin secretion [19, 20]. Glomerular filtration measured by creatinine clearance does not vary according to treatment compared to controls (Table 5), which suggest that the increase in diuresis would rather have a tubular origin as seems to show the clearance of free water (Table 5). It is significantly higher in animals that received the plant extract compared to controls (*P* < 0.05). A single dose of Furosemide 2 mg/kg was administered orally to rats in search of diuretic action on renal or hepatic insufficiency [21]. It acts by inhibiting the reabsorption of Na^+^ and Cl^−^ in the ascending branch of Henle loop. It also has a peripheral and independent renal vascular action [22, 23]. At this level, it inhibits the reabsorption of sodium. It primarily causes urinary sodium excretion and elimination of a significant chloride. It also ensures the tubuloglomerular feedback inhibition without necessary increasing the filtration (Table 5). Major changes would occur in the pore pressure and the interstitial volume and absolute proximal reabsorption remains constant [24].

Amiloride hydrochlorothiazide (14 mg/kg) induces elimination of unneeded water and salt by the kidneys from the body into the urine [25]. It is also active at the inner medullary collectors where it causes an increase in flow and a decrease in reabsorption of water, sodium, and chloride ions. The secretion of potassium is low, which is probably secondary to an increase of potassium. The extract caused a significant increase (*P* < 0.05) in excretion of K^+^ compared to Amiloride hydrochlorothiazide (14 mg/kg), suggesting aqueous extract of the leaves of* Ficus glumosa* does not act as the Amiloride hydrochlorothiazide. It is known that hypervolemia leads to hypertension [1, 26]. The hypotensive activity of* F. glumosa* can then be explained by its diuretic properties.

High sodium level in animals hydrated with the aqueous extract of the leaves of* F. glumosa* (Table 4) could be secondary to the intensity of diuresis resulting from contraction of the plasma compartment. Variations in diuresis may be invoked to explain variations clearance. Indeed, it is assumed that, for a given uremia, clearance and diuresis vary in the same direction, with the reduced clearance for low urinary flow rates being associated with significant reabsorption of urea at the pelvis [27]. The increase of Na^+^, K^+^, and Cl^−^ generated by the aqueous extract of leaves* F. glumosa* caused alkalinization of the urine, showing a strong inhibiting activity of carbonic anhydrase and saluretic. These effects were mainly observed at the dose of 375 dose mg/kg.

In conclusion, the oral administration of a single dose of the aqueous extract of the leaves of* F. glumosa* increased significantly in 24 h urine volume after treatment. In addition, treatment with the aqueous extract of the leaves of* F. glumosa* increased, in a dose dependent manner, the excretion of Na^+^, K^+^, and Cl^−^ and causes a decrease in urine osmolarity. The stability of aldosterone, the absence of correlation with the plasma levels of sodium, and the increased clearance of free water in the animals receiving the aqueous extract show that increased diuresis and moderate natriuresis elevation are of tubular origin.

## Supplementary Material

Obligatory re-absorption (not controlled) is a consequence of the re-absorption by active transport of Na^+^ on the level of the TCP (almost all Na^+^ is reabsorbed on this level) and water follows Na^+^ by osmosis. Optional re-absorption (controlled) is on the level of the distal circumvented tube (TCD) and collecting tube under the control of the antidiuretic hormone or ADH (or vasopressin) secreted by the pituitary gland and indirectly by the aldosterone. The principal substances secreted at the tubular level are K^+^, H^+^, ammonia, creatin, penicillin.ubular secretion allows elimination of certain useless substances or of surplus in blood and the maintenance of the blood pH by the control of the secretion of H^+^. what results by ↑ secretion of ions H^+^ this involves ↑ pH sanguin and ↓ secretion of ions H^+^ what results by ↓ pH sanguin. The re-absorption of water proceeds according to an indirect mode (to control the blood pressure initially), followed by a continuation of action of the system renin-angiotensin.Angiotensin II goes stimulates the corticosurenal. The system renin-angiotensin causes ↑ glomerular filtration what results in the Secretion of renin by the cells of the juxta glomerular apparatus. The Formation of angiotensin II involves the vasoconstriction of the efferent small artery, which results by ↑ blood pressure in the cluster and ↑ filtration. The Secretion of the aldosterone hormone by corticosurenal gland causes ↑ reabsorption of Na^+^ on the level of the collecting tubule, ↑ reabsorption of water (which follows Na^+^ by osmosis), ↑ blood volume, and ↑ blood pressure.

## Figures and Tables

**Table 1:** Effects of the aqueous extract of *F. glumosa *on the urinary volume and excretion of Na^+^, Cl^−^, and K^+^.

Urinary volume and excretion of Na^+^, Cl^−^, and K^+^				
Drugs (mg/kg)	Urinary volume	Na^+^ excretion	Cl^−^ excretion	K^+^ excretion
	(mL/kg/24 h)	(mEq/kg/24 h)	(mEq/kg/24 h)	(mEq/kg/24 h)
Control	249.31 ± 4.93	18.27 ± 1.01	15.12 ± 2.11	19.54 ± 0.85
Extract				
225	292.15 ± 4.69∗∗∗	53.92 ± 2.12∗∗∗	50.87 ± 2.16∗∗∗	37.58 ± 1.51∗∗∗
300	322.34 ± 4.14∗∗∗	104.24 ± 1.26∗∗∗	101.27 ± 1.03∗∗∗	54.29 ± 0.72∗∗∗
375	393.04 ± 4.03∗∗∗	153.78 ± 1.92∗∗∗	150.89 ± 1.42∗∗∗	46.21 ± 2.38∗∗∗
Furosemide (2 mg/kg)	448.17 ± 2.86∗∗∗	95.45 ± 6.45∗∗∗	92.54 ± 6.22∗∗∗	35.99 ± 2.63∗∗∗
Amiloride H. (14 mg/kg)	278.07 ± 6.41∗∗	75.30 ± 2.36∗∗∗	72.34 ± 2.44∗∗∗	25.51 ± 0.88∗∗

Values are means ± SEM, *n* = 5, ∗∗ < 0.01, ∗∗∗ < 0.001, significant difference compared to the control.

**Table 2:** Effects of the aqueous extract of *F. glumosa *on saluretic and natriuretic activity from 24 h of urine.

Drugs (mg/kg)	Saluretic	Natriuretic	CAI	Saluretic index	Natriuretic index	CAI index
	(Na^+^ + Cl^−^)	(Na^+^/k^+^)	Cl/(Na + K)			
Control	33.39 ± 3.12	0.93 ± 1.18	0.45 ± 0.67	1.00	1.00	1.00
Extract						
225	104.79 ± 4.28∗∗∗	1.43 ± 1.40∗	0.48 ± 0.50∗	3.13	1.53	1.07
300	205.51 ± 2.29∗∗∗	1.92 ± 1.75∗∗	0.64 ± 0.44∗∗	6.15	2.06	1.42
375	304.67 ± 3.34∗∗∗	3.33 ± 0.80∗∗∗	0.75 ± 0.42∗∗	9.12	3.58	1.66
Furosemide (2 mg/kg)	187.99 ± 12.67∗∗∗	2.65 ± 2.45∗∗∗	0.70 ± 0.49∗∗	5.63	2.85	1.55
Amiloride H. (14 mg/kg)	147.64 ± 4.80∗∗∗	2.95 ± 2.68∗∗∗	0.48 ± 0.50∗∗	4.42	3.17	1.07

Values are means ± SEM, *n* = 5, ∗ < 0.05, ∗∗ < 0.01, and ∗∗∗ < 0.001, significant difference compared to the control.

CAI: carbonic anhydrase inhibition;

saluretic index: saluretic activity in test group/saluretic activity in control group;

natriuretic index: natriuretic activity in test group/natriuretic activity in control group;

carbonic anhydrase inhibition index: CAI activity in test group/CAI activity in control group.

**Table 3:** Effects of the aqueous extract of *F. glumosa *on urine output index and electrolytic excretion index in 24 hours of urine collection.

Drugs (mg/kg)	Diuretic index	Na^+^ index	Cl^−^ index	K^+^ index	*n*
Control	1.00	1.00	1.00	1.00	5
Extract					
225	1.17	2.95	3.36	1.92	5
300	1.29	5.71	6.70	4.31	5
375	1.57	8.42	9.98	3.90	5
Furosemide (2 mg/kg)	1.79	5.22	6.12	3.38	5
Amiloride H. (14 mg/kg)	1.11	4.12	4.78	1.30	5

*n*: number of animals used in each group.

Diuretic index: urine volume of test group/urine volume of control group.

Na^+^ index: sodium excretion in test group/sodium excretion in control group.

K^+^ index: potassium excretion in test group/potassium excretion in control group.

Cl^−^ index: chloride excretion in test group/chloride excretion in control group.

**Table 4:** Effects of the aqueous extract of *F. glumosa *on the serum parameters.

Drugs (mg/kg)	Glucose (mg/dl)	Creatinine (mg/dl)	Urea (mg/dl)	Albumin (g/l)	Aldosterone (pg/mL)	Na^+^ (méq*·*L^−1^)	K^+^ (méq*·*L^−1^)	POSM (mosmol/kg)
Control	94.39 ± 10.45	0.62 ± 0.41	23.27 ± 4.25	42.8 ± 5.01	293.4 ± 36.23	1.73 ± 0.55	1.85 ± 0.85	262 ± 21
Extract								
225	97.39 ± 10.45∗	0.69 ± 0.73∗	24.49 ± 2.18	43.4 ± 4.11	295.3 ± 28.55	7.79 ± 1.79∗∗∗	3.99 ± 2.82∗∗∗	269 ± 22∗∗
300	96.27 ± 11.22∗	0.72 ± 0.46∗∗	24.55 ± 4.77∗	44.6 ± 5.03∗∗	297.4 ± 29.34∗∗	9.68 ± 1.53∗∗∗	4.89 ± 3.65∗∗∗	270 ± 31∗∗∗
375	98.28 ± 7.10∗	0.89 ± 0.20∗∗∗	25.89 ± 5.26∗∗∗	44.8 ± 6.06∗∗∗	304.25 ± 94.81∗∗∗	10.54 ± 1.92∗∗∗	5.62 ± 3.78∗∗∗	275 ± 42∗∗∗
Furosemide (2 mg/kg)	98.13 ± 6.23∗	0.78 ± 0.22∗∗∗	24.89 ± 4.29∗	44.4 ± 5.13∗∗	303.11 ± 76.77∗∗∗	11.14 ± 1.12∗∗∗	6.34 ± 2.11∗∗∗	276 ± 32∗∗∗
Amiloride H. (14 mg/kg)	97.14 ± 7.21∗	0.76 ± 0.16∗∗∗	26.77 ± 3.95∗∗∗	43.7 ± 4.36∗∗∗	294.45 ± 69.54∗∗∗	7.54 ± 1.33∗∗∗	8.66 ± 3.69∗∗∗	275 ± 44∗∗∗

Values are means ± SEM, *n* = 5, ∗ < 0.05, ∗∗ < 0.01, and ∗∗∗ < 0.001, significant difference compared to the control. POSM: plasma osmolality.

**Table 5:** Effects of the aqueous extract of *F. glumosa *on index kidney function.

Drugs (mg/kg)	Control	225 mg/kg	300 mg/kg	375 mg/kg	Furosemide (2 mg/kg)	Amiloride H. (14 mg/kg)
Creatinine (mg/24 h)	24.66 ± 4.93	17.66 ± 1.52∗∗∗	21 ± 2.64∗∗	25.00 ± 3.60∗	20.00 ± 4.35∗∗	26.33 ± 5.50
CreatC (mL/min)	0.027 ± 0.02	0.024 ± 0.06∗	0.021 ± 0.04∗∗	0.019 ± 0.03∗∗∗	0.020 ± 0.02∗∗	0.019 ± 0.04∗∗∗
Urea (g/24 h)	26.33 ± 3.21	22.33 ± 1.52∗∗∗	23.00 ± 4.35∗	25.66 ± 4.72	23.66 ± 1.52∗∗	21.33 ± 1.52∗∗∗
Uosm (mosmol/kg)	199 ± 17	109 ± 18∗∗∗	111 ± 13∗∗∗	121 ± 17∗∗∗	169 ± 28∗∗∗	157 ± 12∗∗∗
GFR (mL/min)	1.60 ± 0.51	1.53 ± 0.32∗	1.50 ± 0.12∗	1.40 ± 0.14∗∗	1.35 ± 0.17∗∗∗	1.30 ± 0.22∗∗∗
Cosm (mL/min)	0.046 ± 0.005	0.044 ± 0.011	0.045 ± 0.015	0.047 ± 0.007∗∗	0.075 ± 0.011∗∗∗	0.065 ± 0.009∗∗∗
C_H_2_O_. (mL/min)	0.055 ± 0.011	0.053 ± 0.014	0.055 ± 0.021	0.065 ± 0.006∗∗∗	0.067 ± 0.012∗∗∗	0.065 ± 0.014∗∗∗

Values are means ± SEM, *n* = 5, ∗ < 0.05, ∗∗ < 0.01, ∗∗∗ < 0.001, significant difference compared to the control.

CreatC: creatinine clearance; Uosm: urinary osmolarity;

GFR: glomerular filtration rate; Cosm: osmolar clearance;

C_H_2_O_: free water clearance.
