# Supplementary material for: Diuretic Activity of the Aqueous Extract Leaves of Ficus glumosa Del. (Moraceae) in Rats
Source: ScientificWorldJournal. 2014 Oct 14;2014:693803. doi: 10.1155/2014/693803 (PMC4212545; doi:10.1155/2014/693803)
Supplement: Supplementary file 1 — Obligatory re-absorption (not controlled) is a consequence of the re-absorption by active transport of Na+ on the level of the TCP (almost all Na+ is reabsorbed on this level) and water follows Na+ by osmosis. Optional re-absorption (controlled) is on the level of the distal circumvented tube (TCD) and collecting tube under the control of the antidiuretic hormone or ADH (or vasopressin) secreted by the pituitary gland and indirectly by the aldosterone. The principal substances secreted at the tubular level are K+, H+, ammonia, creatin, penicillin.ubular secretion allows elimination of certain useless substances or of surplus in blood and the maintenance of the blood pH by the control of the secretion of H+. what results by ↑ secretion of ions H+ this involves ↑ pH sanguin and ↓ secretion of ions H+ what results by ↓ pH sanguin. The re-absorption of water proceeds according to an indirect mode (to control the blood pressure initially), followed by a continuation of action of the system renin-angiotensin.Angiotensin II goes stimulates the corticosurenal. The system renin-angiotensin causes ↑ glomerular filtration what results in the Secretion of renin by the cells of the juxta glomerular apparatus. The Formation of angiotensin II involves the vasoconstriction of the efferent small artery, which results by ↑ blood pressure in the cluster and ↑ filtration. The Secretion of the aldosterone hormone by corticosurenal gland causes ↑ reabsorption of Na+ on the level of the collecting tubule, ↑ reabsorption of water (which follows Na+ by osmosis), ↑ blood volume, and ↑ blood pressure. [file 693803.f1.doc]

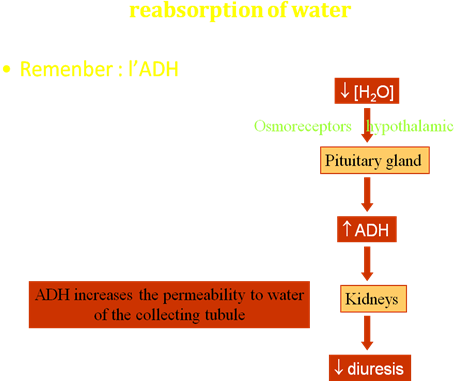


**­ secretion of ions H^+^ ==> ­ pH sanguin**

**↓ secretion of ions H^+^ ==> ↓ pH sanguin**

**1. Obligatory reabsorption (not controlled)**

1. Is a consequence of the reabsorption by active transport of Na^+^ on the level of the TCP (almost all Na ^+^ is reabsorbed on this level)

2. Water follows Na^+^ by osmosis

**2.Optional reabsorption (controlled)**

on the level of the distal circumvented tube (TCD) and tubes collecting, Under the control of the antidiuretic hormone or ADH (or vasopressine) secreted by the hypophyse., and indirectly by the aldosterone

**The reabsorption of water: the aldosterone**

• indirect mode of water reabsorption (to control pressure initially)

• continuation with action of the system renin-angiotensin: Angiotensin II stimulates corticosuprarenal suprarenal, Secretion of the hormone aldosterone by corticosuprarenal gland

The aldosterone causes:

­ ­reabsorption of Na ^+^ on the level of the collecting tubule

==> ­ reabsorption of water (which follows Na ^+^ by osmosis)

==> ­ blood volume

==> ­ blood pressure
